# A Comparison of Intrathecal and Intravenous Morphine for Analgesia After Hepatectomy: A Randomized Controlled Trial

**DOI:** 10.1007/s00268-020-05437-x

**Published:** 2020-02-28

**Authors:** Grzegorz Niewiński, Wojciech Figiel, Michał Grąt, Marta Dec, Marcin Morawski, Waldemar Patkowski, Krzysztof Zieniewicz

**Affiliations:** 1 Department of Anaesthesiology and Intensive Care, Medical University of Warsaw, Warsaw, Poland; 2 Department of General, Transplant, and Liver Surgery, Medical University of Warsaw, 1A Banacha Street, 02-097 Warsaw, Poland

## Abstract

**Background:**

Effective analgesia is essential for patient recovery after liver resection. This study aimed to evaluate the effects of the addition of preoperative intrathecal morphine to multimodal intravenous analgesia in patients undergoing liver resection.

**Methods:**

In this single-blind randomized controlled trial, patients undergoing liver resection were randomly assigned to the patient-controlled analgesia with (ITM-IV) or without (IV) preoperative intrathecal morphine groups. All patients received acetaminophen and dexketoprofen. The primary outcome was pain severity at rest over three postoperative days, assessed using the numerical rating scale (NRS).

**Results:**

The study included 36 patients (18 in each group). The mean maximum daily NRS scores over the first three postoperative days in the ITM-IV and IV groups were 1.3, 1.1, and 0.3 and 1.6, 1.1, and 0.7, respectively (*p* = 0.580). No differences were observed in pain severity while coughing, with corresponding scores of 2.8, 2.1, and 1.1, respectively, in the ITM-IV group and 2.3, 2.2, and 1.5, respectively, in the IV group (*p* = 0.963). Proportions of patients reporting clinically significant pain at rest and while coughing were 11.1% and 44.4%, respectively, in the ITM-IV group, and 16.7% and 44.4%, respectively, in the IV group (both *p* > 0.999). Cumulative morphine doses in the ITM-IV and IV groups were 26 mg and 17 mg, respectively (*p* = 0.257). Both groups also showed similar time to mobilization (*p* = 0.791) and solid food intake (*p* = 0.743), sedation grade (*p* = 0.584), and morbidity (*p* = 0.402).

**Conclusions:**

Preoperative intrathecal morphine administration provides no benefits to multimodal analgesia in patients undergoing liver resection.

**Trial registration number:**

Clinicaltrial.gov Identifier: NCT03620916

## Introduction

Anesthesia for liver resection should be tailored for hemodynamic stability amid hypotension caused by surgical maneuvers or hemorrhage, allowing for rapid emergence and quick recovery with emphasis on adequate pain relief [1]. Many anesthetists favor a combination of general and regional anesthesia. Combined epidural and spinal anesthesia offers patients’ effective intraoperative and postoperative analgesia, while reducing opioid consumption and accelerating bowel remobilization. Furthermore, regional anesthesia decreases respiratory and thromboembolic complication incidences [2–4].

Although thoracic epidural anesthesia (TEA) is used in major abdominal surgery, its safety in postoperative coagulopathy cases has raised concerns [5]. Moreover, TEA may increase transfusion requirements and is associated with relatively high failure rates [6]. In contrast, single-dose intrathecal morphine administration is an easy and viable alternative. It has been successfully used in abdominal surgery for over 20 years [7]. Its advantages over TEA include a lower risk of intraoperative hypotension, reduced fluid requirements, no postoperative motor block, and shorter hospitalization [8]. However, a consensus on use in patients undergoing liver resections is lacking. Therefore, this study evaluates the potential benefits of single-dose intrathecal morphine administration in multimodal analgesia in patients undergoing liver resection.

## Materials and methods

This randomized, single-blind, parallel, controlled trial investigated the effects of intrathecal administration of single-dose morphine in patients undergoing liver resection at the Department of General, Transplant, and Liver Surgery (Medical University of Warsaw). Patients were randomly assigned in a 1:1 ratio to the intrathecal morphine or control group. The primary outcome was the intensity of pain over the first three postoperative days assessed on the numerical rating scale (NRS) at 12-h intervals. Previous studies hypothesized that intrathecal morphine decreases mean maximum NRS score in subsequent assessments on postoperative days 1, 2, and 3 from 4, 4, and 3, respectively, to 2, 2, and 2, respectively [9]. Accordingly, considering the levels of type I and II errors of 0.05 and 0.20, respectively, and NRS standard deviation of 3, the sample size was calculated to be 36 (18 patients in each group). The inclusion criteria were age between 18 and 75 years, provision of informed consent, and liver resection of a suspected malignant tumor. The exclusion criteria were >3 points in the American Society of Anesthesiologists (ASA) scale, intrathecal morphine administration contraindications, chronic preoperative intake of analgesics, history of opioid dependence, body mass index >45 kg/m^2^, and allergy to any analgesic drug administered in the study. The study protocol was approved by the institutional review board of the Medical University of Warsaw. All patients provided informed consent prior to enrollment. The study was preregistered in the international registry clinicaltrials.gov (NCT03620916). Patients were screened for eligibility and enrolled in the study between August 17, 2018, and January 10, 2019. Follow-up was closed on April 10, 2019.

Pain severity, reflected by the maximum NRS scores at rest on postoperative days 1, 2, and 3, was the primary outcome. The secondary outcomes included the maximum NRS scores while coughing on postoperative days 1, 2, and 3; total dose of morphine administered intravenously and subcutaneously over the first three postoperative days; time to patient mobilization, indicated by standing unassisted; grade of patient sedation; time to first solid food intake tolerance; hospitalization duration; and postoperative complications.

Randomization was performed using sealed envelopes containing computer-generated intervention codes, which were drawn by the anesthesiologist immediately before surgery within the theater. Although the patients were aware of the assignment, the surgeons, other care providers, and investigators evaluating outcome measures were blinded. Patients in the intrathecal morphine group received single-dose intrathecal morphine (0.4 mg diluted to 4 mL in 0.9% solution of sodium chloride) through lumbar puncture at the level of L3/L4 or L4/L5 with a 26-gauge needle immediately before anesthesia induction and no intravenous morphine before the cessation of anesthesia. Patients in the control group received single-dose intravenous morphine (0.15 mg/kg of body weight) 30 min before extubation. Postoperatively, morphine was intravenously administered in both groups via patient-controlled analgesia (PCA) at doses of 2 mg with at least 20-min intervals for 24 h. Subsequently, when NRS scores were >4, 5 mg of morphine was administered subcutaneously with at least 6-h intervals. Additionally, all patients received paracetamol (1.0 g every 6 h) and dexketoprofen (50 mg every 8 h). Antiemetic prophylaxis included single-dose intravenous dexamethasone (4 mg) and ondansetron (4 mg) administered during the operation. Postoperative antiemetic prophylaxis was not used; the patients received ondansetron (4 mg) intravenously exclusively for nausea or vomiting. All patients received oral midazolam (7.5 mg) premedication approximately 30 min before anesthesia. Induction of general anesthesia comprised intravenous propofol (2 mg/kg), remifentanil (0.1 µg/kg/min), and cisatracurium (0.1 mg/kg). General anesthesia was maintained using desflurane, cisatracurium, and remifentanil. After anesthesia induction, remifentanil was administered continuously at 0.05–0.1 µg/kg/min with additional boluses of 0.5 µg/kg in cases of systolic hypertension or tachycardia of 20% or more, with clinical assessment indicating pain as the cause.

Liver resections were performed through bilateral subcostal incisions. The Pringle maneuver was used selectively. Parenchymal transection was performed using an ultrasonic device. Abdominal drains were routinely left near the transection planes. Continuous loop polydioxanone sutures were used for fascial closure. Skin closure was performed either using staples or interrupted sutures. Major resections were defined as the removal of over two liver segments.

Pain severity was assessed at rest and while coughing at 10 p.m. on postoperative day 0 and at 10 a.m. and 10 p.m. on postoperative days 1, 2, and 3. The total dose of morphine administered through PCA and subcutaneously, on postoperative days 0, 1, 2, and 3, was noted. All patients were strictly followed up for the occurrence of potential complications of intrathecal morphine administration and postoperative complications for 90 postoperative days. Complications were graded using the Clavien–Dindo classification. Grade of sedation was assessed using the Richmond Agitation–Sedation Scale.

Quantitative variables were presented as medians with interquartile ranges or means with standard errors, depending on their distribution. The Shapiro–Wilk test was used for assessing normal distribution. Qualitative variables were presented as numbers with frequencies. Intergroup comparisons of quantitative variables were performed using the Mann–Whitney *U* test for non-normally distributed variables or the *t* test for normally distributed variables. Intergroup comparisons of qualitative variables were performed using Fisher’s exact test. Analysis of variance for repeated measurements was used to compare NRS scores between groups in the postoperative period. The level of significance was set at a two-tailed *p* of 0.05. Statistica version 13 [TIBCO Software Inc. Palo Alto, CA, USA (2017)] was used for statistical analyses.

## Results

**Fig. 1:**
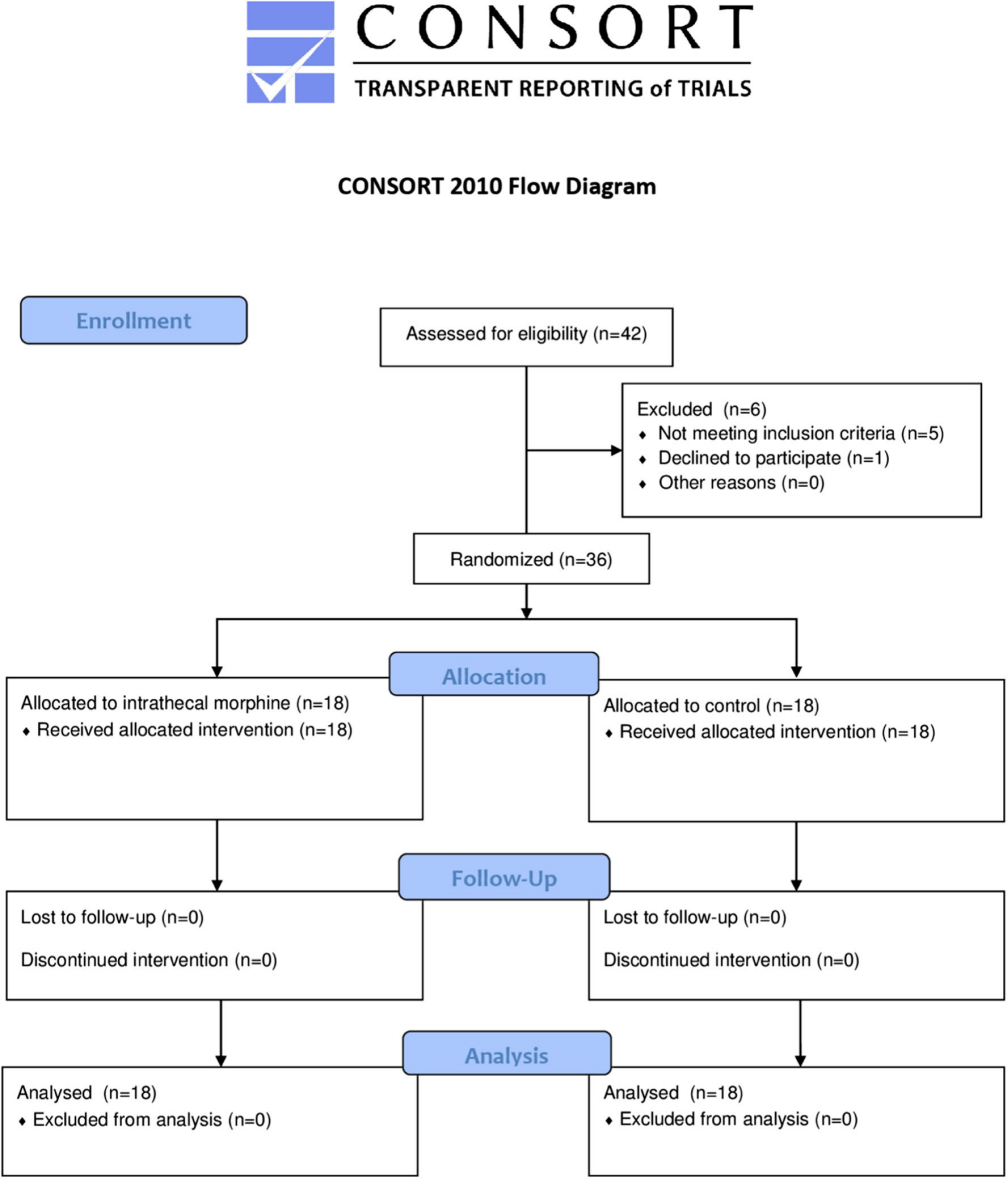
CONSORT flow diagram

Out of 42 patients screened for eligibility, 36 were included in the study. Among them, 18 each were assigned to the intrathecal morphine and intravenous morphine groups (Fig. 1). Overall characteristics of the study cohort and intergroup comparisons are presented in Table 1. Patients receiving intrathecal morphine were younger, had remarkably lower ASA scores, and underwent major resections less frequently. Regarding indications for surgery, primary liver malignancies were less frequent in the intrathecal morphine group. Otherwise, both groups had similar baseline characteristics.

**Table 1:** Baseline characteristics of the study cohort and comparisons between patients in the intrathecal morphine group and control group

Characteristics	All patients (*n* = 36)	Intrathecal morphine (*n* = 18)	Control (*n* = 18)	*p*
Patient sex				0.500
Male	15 (41.7%)	9 (50.0%)	6 (33.3%)	
Female	21 (58.3%)	9 (50.0%)	12 (66.7%)	
Patient age (years)	58 (54–67)	56 (47–62)	63 (58–68)	0.012
Weight (kg)	75 (64–87)	77 (62–92)	72 (65–86)	0.937
Height (cm)	167 (164–176)	169 (164–177)	165 (162–175)	0.518
Body mass index (kg/m^2^)	25.9 (23.3–29.2)	26.1 (22.5–29.0)	25.9 (24.0–31.3)	0.773
Indication for resection				0.054
Colorectal metastases	14 (38.9%)	8 (44.4%)	6 (33.3%)	
Primary malignancies	10 (27.7%)	2 (11.1%)	8 (44.4%)	
Extrahepatic biliary and gallbladder malignancies	5 (13.9%)	2 (11.1%)	3 (16.7%)	
Other	7 (19.4%)	6 (33.3%)	1 (5.6%)	
Comorbidities				
Diabetes	3 (8.3%)	1 (5.6%)	2 (11.1%)	>0.999
Arterial hypertension	15 (41.7%)	6 (33.3%)	9 (50.0%)	0.500
COPD	1 (2.8%)	0 (0.0%)	1 (5.6%)	>0.999
Laboratory tests				
WBC (10^3^/mm^3^)	6.7 (5.4–8.0)	6.1 (5.3–7.4)	6.8 (5.8–8.0)	0.207
Hemoglobin (g/dL)	13.1 (12.0–13.8)	13.3 (12.4–13.7)	13.1 (12.0–13.8)	0.481
Platelets (10^3^/mm^3^)	228 (191–269)	225 (177–251)	233 (201–313)	0.389
Bilirubin (mg/dL)	0.4 (0.3–0.7)	0.4 (0.3–0.6)	0.4 (0.4–0.7)	0.293
INR	1.0 (1.0–1.1)	1.0 (1.0–1.1)	1.0 (1.0–1.1)	0.912
Albumin (g/dL)	4.4 (4.2–4.6)	4.4 (4.1–4.5)	4.4 (4.2–4.6)	0.542
ASA score				0.064
I	13 (36.1%)	9 (50.0%)	4 (22.2%)	
II	16 (44.4%)	8 (44.4%)	8 (44.4%)	
III	7 (19.4%)	1 (5.5%)	6 (33.3%)	
Major liver resection	19 (52.8%)	7 (38.9%)	12 (66.7%)	0.181
Duration of surgery (min)	208 (150–263)	235 (155–275)	190 (150–250)	0.606
Duration of anesthesia (min)	250 (195–315)	275 (190–335)	225 (200–290)	0.601
Blood loss (mL)	300 (200–450)	300 (200–400)	350 (200–500)	0.606
Total intraoperative fluid administration (L)	2.6 (2.3–3.1)	2.5 (2.3–3.0)	2.7 (2.3–3.2)	0.839
Total intraoperative remifentanil dose (mg)	1.3 (1.1–1.6)	1.5 (1.1–1.6)	1.2 (0.9–1.5)	0.279

Data are presented as medians with interquartile ranges or numbers with percentages. A comparison of continuous variables was performed using the Mann–Whitney *U* test or *t *test, depending on their distribution

*COPD* chronic obstructive pulmonary disease, *WBC* white blood count, *INR* international normalized ratio, *ASA* American Society of Anesthesiologists

**Fig. 2:**
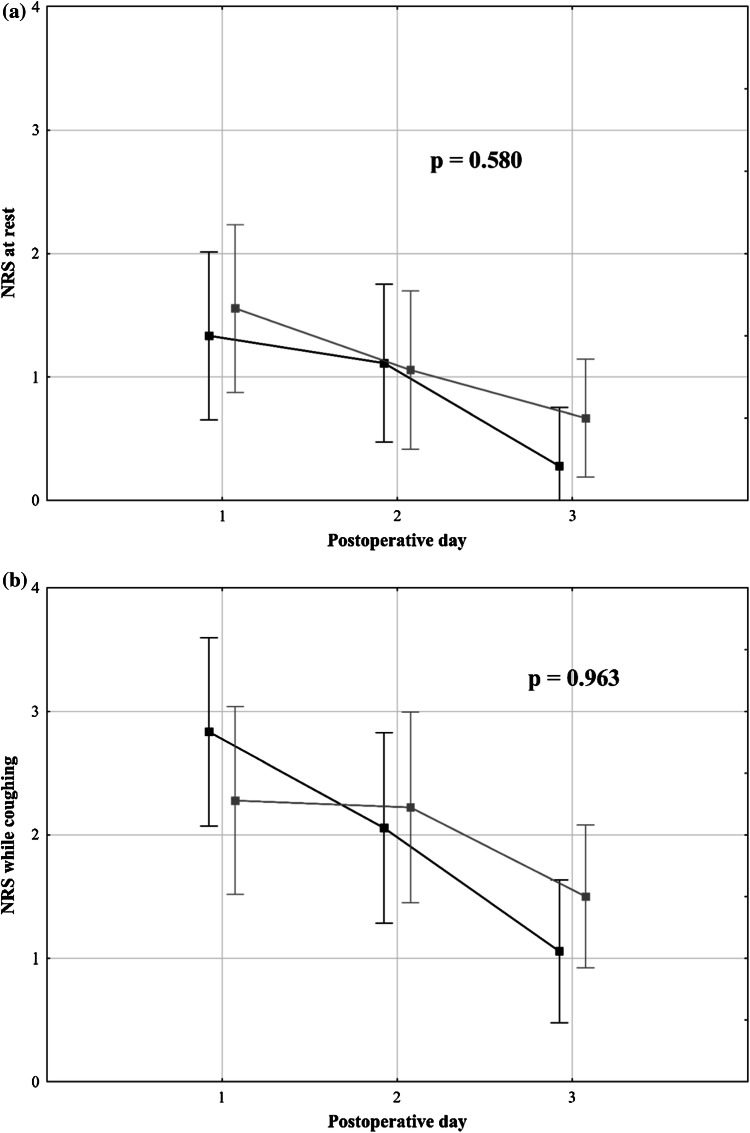
Mean maximum daily numerical rating scale scores at rest (**a**) and while coughing (**b**), with standard errors in patients after liver resection with (black) and without (gray) preoperative intrathecal morphine administration

**Fig. 3:**
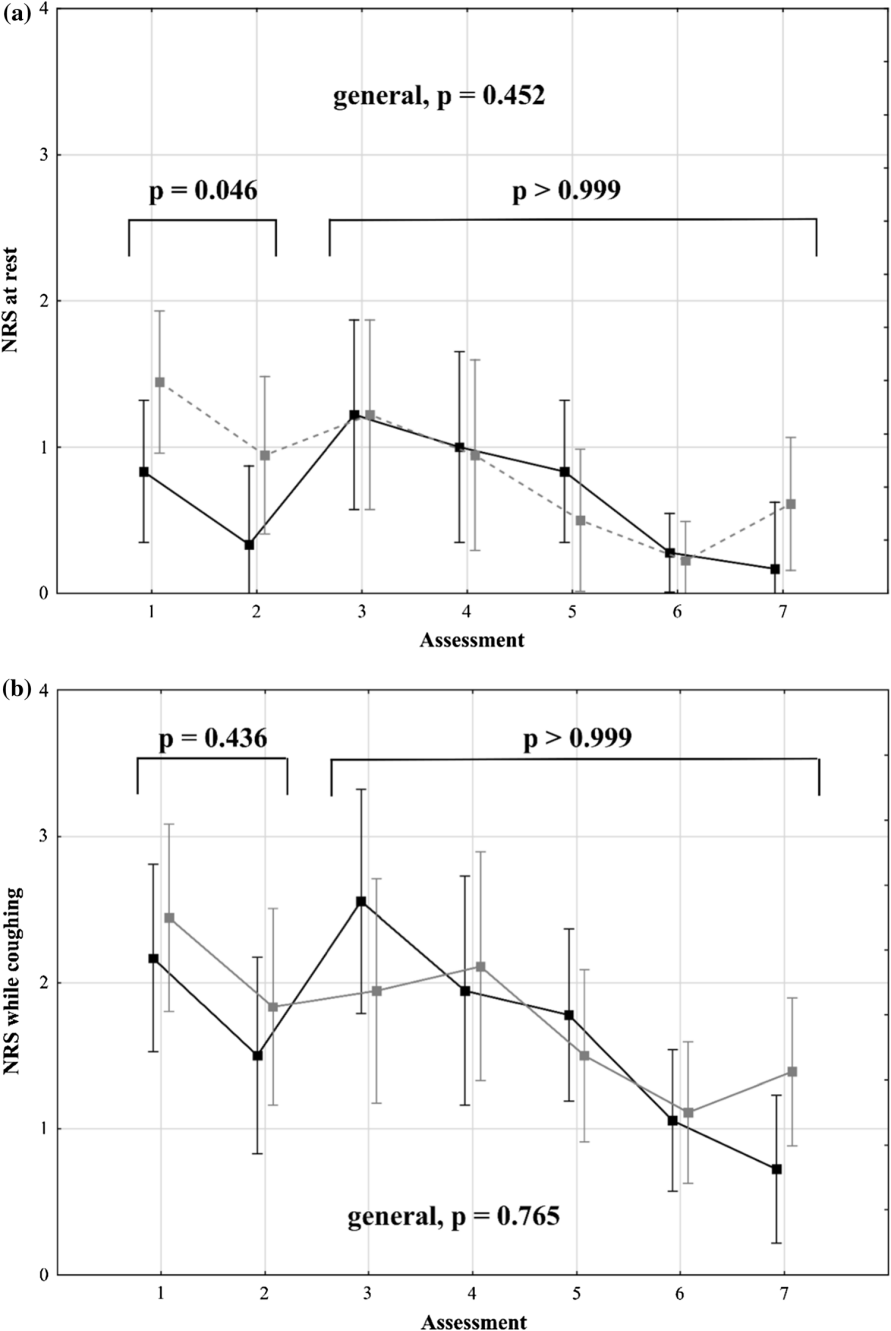
Mean numerical rating scale scores at rest (**a**) and while coughing (**b**) with standard errors in subsequent assessments at 12-h intervals in patients after liver resection with (black) and without (gray) preoperative intrathecal morphine administration

Generally, no significant intergroup difference was observed in pain severity over the first three postoperative days. The mean maximum daily NRS scores at rest on the first, second, and third postoperative days were 1.3 (0.3), 1.1 (0.3), and 0.3 (0.2), respectively, in the intrathecal morphine group and 1.6 (0.4), 1.1 (0.3), and 0.7 (0.3), respectively, in the control group (*p* = 0.580; Fig. 2a). Generally, no difference was observed in all NRS scores at rest on days 0, 1, 2, and 3 evaluated at 12-h intervals (*p* = 0.452; Fig. 3a). However, patients in the intrathecal morphine group had significantly lower NRS scores at rest at the first two assessments within 24 h after the operation, with no difference afterward (*p* > 0.999). The mean NRS scores at rest at the first two evaluations over the 24-h postoperative period were 0.8 (0.2) and 0.3 (0.2) in the intrathecal morphine group and 1.4 (0.2) and 0.9 (0.3) in the control group (*p* = 0.046).

The mean maximum daily NRS scores while coughing on the first, second, and third postoperative days were 2.8 (0.4), 2.1 (0.3), and 1.1 (0.3), respectively, in the intrathecal morphine group and 2.3 (0.4), 2.2 (0.4), and 1.5 (0.3), respectively, in the control group (*p* = 0.963; Fig. 2b). Regarding all postoperative NRS scores while coughing, no significant intergroup differences were observed (*p* = 0.765) over the first two assessments during the 24-h postoperative period (*p* = 0.436), and during the subsequent period (*p* > 0.999; Fig. 3b).

The median postoperative hospitalization duration was 6.5 and 7 days in the intrathecal morphine and control groups, respectively (*p* = 0.044). No significant differences were observed in other secondary outcomes, namely total dose of morphine administered intravenously and subcutaneously over the first three postoperative days (*p* = 0.257), time to patient mobilization as indicated by standing unassisted (*p* = 0.791), patient’s sedation grade on the day of surgery (*p* = 0.584) and the first postoperative day (*p* = 0.424), time to solid food intake tolerated (*p* = 0.743), and postoperative complications (*p* = 0.402; Table 2). Both groups also showed similar times to sitting with assistance and sitting unassisted, time to standing with assistance, times to walking with assistance and walking unassisted, time to oral water intake, and times to first flatus and defecation. The rate of postoperative nausea and vomiting was 16.7% (3 of 18) in the intrathecal morphine group and 38.9% (7 of 18) in the control group (*p* = 0.264). Notably, the number of patients experiencing clinically significant pain (NRS score ≥ 4) at rest and while coughing did not differ between the two groups (both *p* > 0.999). No patient experienced any potential complications directly associated with lumbar puncture and intrathecal morphine administration, such as headache, paresthesia, or lower limb muscle strength impairment. Moreover, none of the patients reported pruritus. No episodes of hypotension or respiratory depression related to intrathecal morphine administration were observed. Urinary catheters were maintained until the morning of postoperative day 1, and no urinary retention episodes were noted in any patient.

**Table 2:** Comparison of patients in the intrathecal morphine group and the control group concerning secondary outcome measures and other (not prespecified) outcome measures

Outcomes	Intrathecal morphine (*n* = 18)	Control (*n* = 18)	*p*
*Secondary outcome measure*			
Total dose or morphine over first three postoperative days (mg)	26 (10–44)	17 (10–28)	0.257
Time to patient mobilization (days)	1 (1–2)	1 (1–2)	0.791
*RASS*			
Day 0	−1 (−1 to 0)	−1 (−1 to 0)	0.584
Day 1	0 (0–0)	0 (0–0)	0.424
Time to solid food intake (days)	2 (1–3)	2 (1–3)	0.743
Duration of postoperative hospitalization (days)	6.5 (5–7)	7 (6–10)	0.044
Complications (≥3 Clavien–Dindo grade)	2 (11.1%)	5 (27.8%)	0.402
*Other (not prespecified) outcome measures*			
Any episode of NRS ≥4			
At rest	2 (11.1%)	3 (16.7%)	>0.999
While coughing	8 (44.4%)	8 (44.4%)	>0.999
Time to sitting with assistance (days)	1 (1–1)	1 (1–1)	0.462
Time to sitting alone (days)	1 (1–2)	1 (1–1)	0.563
Time to standing with assistance (days)	1 (1–2)	1 (1–1)	0.791
Time to walking with assistance (days)	1 (1–2)	1 (1–2)	0.791
Time to walking alone (days)	1 (1–2)	2 (1–2)	0.443
Time to oral water intake (days)	1 (1–1)	1 (1–1)	0.462
Time to first flatus (days)	2 (2–3)	2 (2–3)	0.864
Time to first defecation (days)	3.5 (3–5)	3 (3–4)	0.462
Postoperative nausea or vomiting	3 (16.7%)	7 (38.9%)	0.264

Data are presented as medians with interquartile ranges in brackets or as numbers with percentages in brackets

*RASS* Richmond Agitation–Sedation Scale

## Discussion

Optimal analgesia is critical to patient recovery after liver resection. The two forms of neuraxial analgesia, namely epidural analgesia and intrathecal morphine administration, are effective in reducing pain severity in the postoperative period in patients undergoing hepatobiliary operations [9–12]. In this randomized trial, intrathecal morphine administration showed no clinically relevant benefits. Considering the 3-day postoperative period, the only significant difference between patients receiving intrathecal morphine and PCA was slightly lower pain severity in the former, despite lower mean NRS scores (1.4 and 0.9) in the latter. Further, the number of patients reporting any episode of clinically significant pain (NRS score ≥ 4) was almost identical in both groups. These findings contradict previously published results.

In previous studies proving the superiority of preoperative intrathecal morphine in patients undergoing hepatobiliary operations, pain severity was apparently higher both in patients with and without intrathecal morphine administration [9–11]. These three studies included a similar number of patients who received similar doses of the intrathecal regimen comprising 0.5 mg of morphine, 15 μg of fentanyl, 0.4 mg of morphine, and 4 μg/kg of morphine. Based on these three previous studies demonstrating a positive effect of intrathecal morphine, we selected a dose of 0.4 mg as being low and potentially effective. The only clinically remarkable difference between this and the previous studies is, however, the use of multimodal analgesia. In this study, all patients routinely received dexketoprofen and paracetamol at regular intervals irrespective of the reported pain severity. In contrast, non-opioid analgesic drugs were either not given or given only in case of severe pain, despite maximal dosing of opioids. Accordingly, the extremely low NRS scores observed in the present study are likely due to regular preemptive dexketoprofen and paracetamol administration, regardless of reported pain severity. The cumulative dose of opioids administered in the postoperative period was several folds higher than those administered in previous studies, even in patients assigned to the intrathecal morphine group [9–11]. No significant reduction in postoperative morphine administration was observed in patients receiving intrathecal morphine in the present study, unlike in previous studies. Although the cumulative doses of morphine administered over three postoperative days were low at the median levels of 26 mg and 17 mg, pain control was adequate in both groups. In contrast, one of the previous studies was prematurely terminated because of extremely high morphine consumption in patients not receiving intrathecal morphine [10]. Therefore, although the present study was not designed to assess the effects of multimodal analgesia after liver resection, its findings suggest there are no benefits of intrathecal morphine administration when utilizing a multimodal analgesic regimen.

A recent randomized trial indicated the superiority of epidural analgesia over intravenous analgesia for pain control in the postoperative period after liver resection [12]. However, non-opioid analgesics were used only at the discretion of the attending physicians and pain severity reported by patients in both groups was remarkably higher than that reported in the present study. In fact, a subsequent randomized trial provided evidence for the non-inferiority of intravenous PCA to epidural analgesia in patients receiving multimodal analgesia comprising routine acetaminophen and ketorolac administration after open liver resections [13]. The lack of benefits of intrathecal morphine administration over intravenous PCA in patients receiving multimodal analgesia contradicting previous findings resembles the contrast between the previous studies on epidural analgesia and the present non-inferiority trial. Previous comparisons of intrathecal and epidural analgesia revealed their similar analgesic efficacy [14–16]. However, the results of this study are limited to intrathecal morphine administration and indicate that utilizing multimodal regimens after liver resection is unnecessary. A non-inferiority trial comparing PCA to intrathecal morphine administration in the context of the present findings is warranted to confirm these findings.

Both intrathecal analgesia and epidural analgesia were previously reported to provide benefits exceeding superior pain control and reduced cumulative dose of opioids. These include less time to patient mobilization and dietary intake, lower rate of general complications, and reduced mortality [14, 15, 17]. On the other hand, epidural analgesia was associated with hypotension, increased use of vasopressors, and impaired kidney function, and intrathecal analgesia was associated with pruritus and late respiratory depression [12, 14, 16, 18–21]. The present study was neither powered nor designed to detect such effects; however, no effects on patient recovery after liver resection and no procedure-specific complications were observed. The only significant difference between groups regarding secondary outcomes was the shorter duration of hospitalization in patients in the intrathecal morphine group. However, as no clinically relevant effects of intrathecal morphine were found with respect to pain management, opioid consumption, restoration of gastrointestinal function, and patient mobilization, this was most probably due to differences in baseline characteristics. Patients in the intrathecal morphine group were younger, had lower ASA scores, and less frequently underwent major resections. The cumulative effects of these differences seem to be the most probable explanation for their shorter period of postoperative hospitalization.

Among the other potential analgesic measures, various forms of local analgesia were previously examined in patients undergoing liver resection. A comparison of intrathecal morphine with ropivacaine wound infusion revealed their similar analgesic efficacy [22]. Perioperative nerve block combined with or without local anesthetic wound infiltration also had comparable efficacy to epidural analgesia in pain management [23, 24]. However, only one study compared local anesthetic wound infusion through medial open transversus abdominis plane catheters to intravenous analgesia and reported less opioid consumption, lower pain scores, and shorter hospital stay in patients receiving the former [25]. However, the multimodal intravenous regimen comprised only a combination of an opioid with a non-opioid drug, and in fact, both opioid consumption and pain scores were remarkably higher in both the treatment and placebo groups than those reported by patients in the present study. Therefore, despite these promising results, the present findings warrant further assessment of the efficacy of the methods of local analgesia in patients receiving a combination of opioids with two non-opioid agents.

This study had several limitations. First, the sample size was calculated to detect differences in higher NRS scores that were observed in the present study; thus, it was not powered to detect small differences in low NRS scores. However, these seem clinically irrelevant. Second, the study was not powered to detect differences in secondary outcomes; nevertheless, considering the lack of a significant impact of intrathecal morphine administration on pain management and cumulative opioid dose, the differences seem unlikely. Further, in this study, only the outcome assessors and attending physicians in the postoperative wards were blinded; neither the anesthesiologist performing intrathecal injection nor the patients were blinded. Nevertheless, the authors decided not to perform sham intrathecal injections for ethical reasons. Finally, the observed insignificantly lower rate of postoperative nausea and vomiting in the intrathecal morphine group was clearly in contrast to the insignificantly higher cumulative opioid dosage. This finding has no clear explanation; however, given the lack of significance, this may be an accidental finding.

In conclusion, the present study provides no evidence for the benefits of preoperative intrathecal morphine administration in patients receiving multimodal analgesia with a combination of morphine and two non-opioid agents after liver resection. Therefore, intrathecal analgesia in patients undergoing liver resection seems unnecessary.
